# Peripheral Vascular Trauma in Pediatrics: A Case Report and Literature Review

**DOI:** 10.7759/cureus.5744

**Published:** 2019-09-24

**Authors:** Jatnna E Soto, Dulce M Vásquez, George Rodríguez, Luis A De La Cruz

**Affiliations:** 1 Department of Pediatrics, Hospital Infantil Dr. Robert Reid Cabral, Santo Domingo, DOM; 2 Cardiovascular Surgery, Hospital Infantil Dr. Robert Reid Cabral, Santo Domingo, DOM

**Keywords:** radial artery, pediatrics, peripheral vascular trauma, radial artery ligation

## Abstract

Vascular lesions constitute an important cause of morbidity and mortality, including functional disability. Their poor documentation in the literature suggests a low frequency in the pediatric age range. Herein, we describe the case of an 11-year-old-boy with an isolated radial artery injury. The patient initially presented with active bleeding from a lacerated wound. He was intervened on multiple occasions for hematoma formation and suture dehiscence at a primary care center from his community. During his admission, he required a blood transfusion for active bleeding. Vascular exploration was performed, and radial artery lesion was managed by ligation.

## Introduction

Less than five cases treated over one year in major trauma hospitals in the United States present with vascular lesions. These can be of iatrogenic or traumatic origin, which, in turn, are classified as contusive or penetrating [1]. The best prognosis is obtained with a high index of suspicion, imaging studies and early surgical intervention [2]. Their poor documentation in the literature suggests a low frequency in the pediatric age range, being caused, in most cases, by penetrating wounds [3]. In some studies, those produced in the forearm (radial/ulnar artery) represent less than 15% of all vascular lesions [1]. The diagnosis in children is complex and may be delayed due to the masking caused by vasospasm. Early diagnosis is vital to ensure a good prognosis. Due to the rarity of these injuries and the lack of standardized guidelines, management is dictated by the particular experience of each center.

## Case presentation

An 11-year-old male from Pedernales was brought to the emergency department with the complaint of a cutting wound with active bleeding on the right forearm. Medical history, family history and the use of medications or anticoagulants were denied. The mother disclosed that the wound occurred accidentally while the child was playing with a pair of scissors. She reported that as soon as the incident happened, approximately a month ago, the patient was taken to a community health center where the wound was managed by primary closure. After seven days, he was taken back to the community center complaining of a mass, consistent with a hematoma, and suture dehiscence. On that occasion, the hematoma was drained and the wound was sutured for the second time. Two weeks after the second intervention, he was brought for the third time to the same health center for drainage of hematoma, suture of the wound and placement of a compression elastic bandage. Due to the lack of improvement and evidence of heavy bleeding, he was referred to our center and admitted for management purposes with the diagnosis of a lacerated wound on the right forearm and probable blood dyscrasia (hemophilia).

On physical examination, he presented with marked pale skin and mucous membranes, especially at the conjunctival level, along with dry oral mucosa. Vital signs were stable. Upper extremities were asymmetric at the expense of the lacerated wound on the volar aspect of the right forearm sutured with nylon thread, with the presence of active bleeding, non-pulsatile hematoma, conserved peripheral pulses and absence of signs of inflammation. He was admitted to the inpatient room for assessment of hydration, vital signs monitoring and laboratory testing. The following results were reported: erythrocytes: 3.65x10^6^/mm^3^, hemoglobin: 9.6 g/dL, hematocrit: 28%, mean corpuscular volume: 77µm^3^, mean corpuscular hemoglobin: 26.5 pg, platelets: 362x10^3^/mm^3^, leukocytes: 10.9x10^3^/mm^3^, granulocytes: 7.6x10^3^/mm^3^, lymphocytes: 2.6x10^3^/mm^3^, glucose: 120 mg/dL, lactate dehydrogenase: 780 U/L. Coagulation tests-prothrombin time, partial thromboplastin time, bleeding time and coagulation time-were normal.

The patient was consulted with the Department of Vascular Surgery and, based on the absence of hard signs of vascular injury during the physical examination, the placement of an elastic bandage with medium compression, immobilization of the limb, and arterial and venous Doppler of the right forearm were recommended. Two days later, the Doppler could not be performed because the technician reported heavy bleeding when removing the elastic bandage. Hence, the patient was administered tranexamic acid 660 mg IV every 24 hours. On the fourth day of admission, he was reassessed by the vascular surgeon and the diagnosis of probable vascular injury was added. Given the evidence of hemodynamic instability, it was decided to transfuse packed red blood cells and perform pre-anesthetic evaluation in the event of a surgical intervention. Afterwards, he was transferred to the operating room for vascular exploration. Under inhalational anesthesia, the suture was removed, blood clots were evacuated and a 90%-95% radial artery lesion was observed. Proximal and distal control was obtained, and an intact palmar arch patency was determined. The radial artery was ligated due to hemodynamic instability. The postoperative course was uneventful and the patient was discharged under stable general conditions. A follow-up appointment was scheduled in the Department of Vascular Surgery as an outpatient. 

## Discussion

Peripheral vascular lesions are grouped into three major classifications: iatrogenic, which occur as part of a diagnostic or therapeutic procedure; those that originate from a blunt trauma that affects the intimal layer; and those resulting from a penetrating trauma that involves partial or complete transection of the blood vessel [1,3]. Penetrating forearm injuries are considered a subtype within the ones that affect the upper limb [4]. In some studies, lesions of the forearm affecting either radial or ulnar arteries represent less than 15% of all vascular injuries [1]. These figures vary considerably, and some authors have reported the penetrating mechanism being more frequent than blunt [5]. Penetrating traumas in the upper limb are mostly caused by pieces of broken glass, knives and sharp instruments [4,6,7]. These are considered as complicated lesions and represent a diagnostic and therapeutic challenge due to the anatomical and physiological characteristics of the pediatric age, such as the configuration of the vessels, which tend to be smaller and thin walled [1].

Initial management involves the control of the bleeding site. For this purpose, direct digital pressure is considered a safe and effective method if applied for a minimum of five minutes without interruption. The use of pneumatic tourniquets at the brachial or antebrachial level is also effective, being applied in children with a pressure between 100 and 200 mmHg, and the technique can also be used with the help of a blood pressure cuff [8]. It should be pointed out that many children with vascular lesions may not show any signs of hemodynamic instability, even after significant blood loss [9]. Immediate surgical exploration is not necessary in cases where the bleeding is controlled by compression maneuvers and a thorough examination of the limb is performed [8].

**Figure 1:**
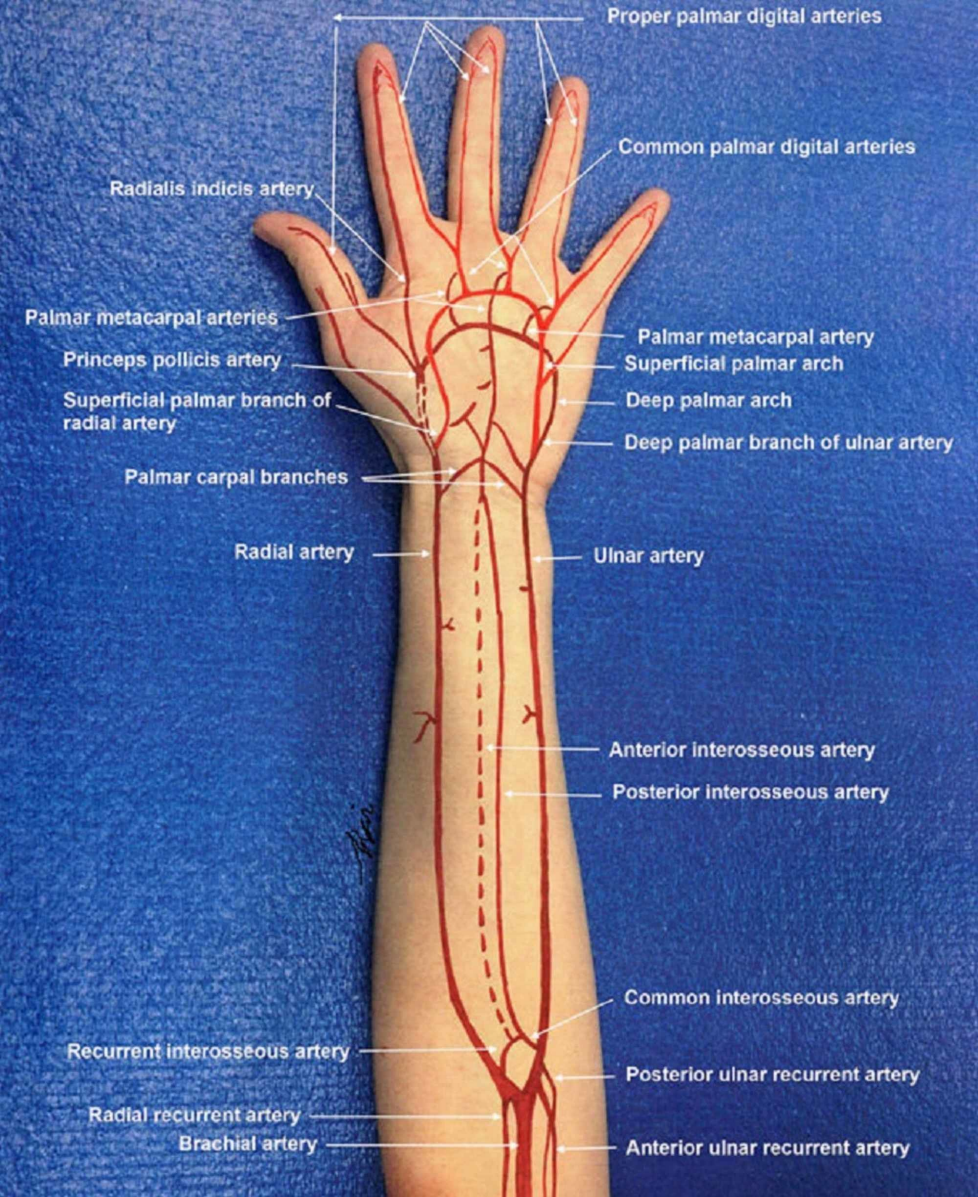
Arterial circulation in the forearm and hand. The radial artery is one of two major blood vessels that supply blood to the forearm and the hand [8].

The diagnosis of peripheral vascular injuries is obtained by a systematic and judicious physical examination. At the forearm level, it may be more evident due to the superficial location of the arteries and the presence of hard signs, such as the absence of a distal pulse, auscultation of a murmur or palpating a thrill, as well as paleness and coldness of the limb (Figure 1) [7,8]. In those cases, Doppler ultrasonography and arteriography are indicated, since these signs are related to a high suspicion of vascular injury. It is important to clarify that the presence of active bleeding does not contraindicate Doppler ultrasound, unless the patient presents with massive bleeding or hemodynamic instability, in which case immediate vascular exploration is indicated. Other hard signs, such as a visible expansive hematoma and pulsatile bleeding, require immediate surgical exploration as well due to the potential risk of complications, including limb ischemia. 

On the other hand, soft signs-neurological deficit, history of significant bleeding, decreased pulse, bone or penetrating injury near an artery-involve observation, imaging studies and ankle-brachial index to rule out the presence of vascular injury [10].

As these are infrequent injuries, there is no documentation on standardized management protocols, which leads to a great variability, evidenced in the reports contained in the literature [9,11]. Depending on the institution, they can be treated by the general surgeon, the pediatric surgeon, the vascular surgeon, orthopedic surgeon or plastic surgeon [11]. Currently used techniques have been derived from the experience with adult patients, and opting for vascular repair or ligation remains at the discretion of the surgeon [2,8,12,13]. Penetrating trauma tends to produce a vascular transection, being more prone to repair in contrast to a closed trauma, which causes a more extensive and disseminated lesion that results inadequate for primary repair [13].

**Figure 2:**
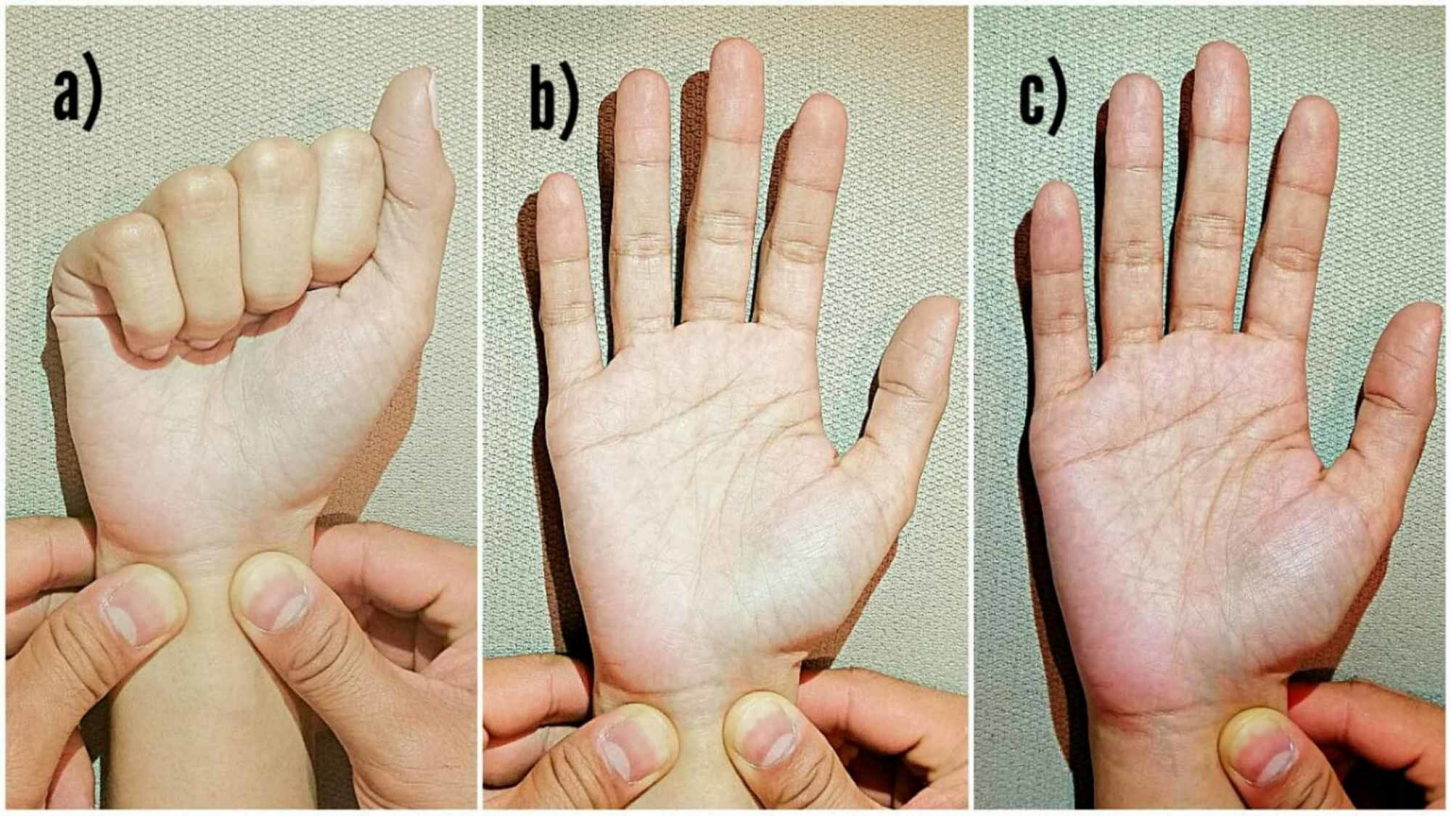
Allen’s test to determine the patency of the palmar arch. a) Radial and ulnar arteries occluded while the patient makes a fist. b) Note the blanching of the palm. c) Ulnar artery is released and capillary refill on the hand is noted.

Even when the treatment of choice is the repair of the blood vessel, the ligation of an isolated radial or ulnar artery does not determine the appearance of ischemia in the limb and is considered a safe procedure that should only be performed after determining the presence of an intact palmar arch (Figure 2) [8].

In some cases, ligation is preferred over repair due to the longer surgical and anesthetic time that the latter entails. However, no significant differences in prognosis and long-term complications have been demonstrated. A favorable recovery has been obtained in both cases, without altering the function of the limb, including the cases treated with ligation [12,14].

## Conclusions

Traumatic vascular lesions in the pediatric population are a very rare entity. The success of the treatment requires a high index of suspicion that allows an early diagnosis and prompt surgical management, in the cases that require it. We emphasize the importance of diagnostic protocols focused on identifying hard and soft signs of vascular injury, especially in community and primary care centers, to warrant a rapid intervention and avoid the development of complications.

## References

[1] Mommsen P, Zeckey C, Hildebrand F (2010). Traumatic extremity arterial injury in children: epidemiology, diagnostics, treatment and prognostic value of Mangled Extremity Severity Score. J Orthop Surg.

[2] Shah SR, Wearden PD, Gaines BA (2009). Pediatric peripheral vascular injuries: a review of our experience. J Surg Res.

[3] Ammar AA (2016). Peripheral arterial injuries in pediatric age group. J Trauma Inj.

[4] Kayssi A, Metias M, Langer JC (2018). The spectrum and management of noniatrogenic vascular trauma in the pediatric population. J Pediatr Surg.

[5] Corneille MG, Gallup TM, Villa C (2011). Pediatric vascular injuries: acute management and early outcomes.. J Trauma Inj Infect Crit Care.

[6] Jaipuria J, Sagar S, Singhal M (2014). Paediatric extremity vascular injuries - experience from a large urban trauma centre in India. Injury.

[7] Wolosker N, Guimarães PC, Gaudêncio A (1994). Trauma to arteries of the forearm. Sao Paulo Med J.

[8] Thai J, Pacheco J, Margolis D (2015). Evidence-based comprehensive approach to forearm arterial laceration. West J Emerg Med.

[9] Kirkilas M, Notrica DM, Langlais CS, Muenzer JT, Zoldos J, Graziano K (2016). Outcomes of arterial vascular extremity trauma in pediatric patients. J Pediatr Surg.

[10] (2019). Extremity vascular trauma workup: approach considerations, laboratory studies, imaging studies. https://emedicine.medscape.com/article/462752-workup.

[11] Gurien LA, Maxson RT, Dassinger MS, Mehl SC, Saylors ME, Smith SD (2017). Pediatric vascular injuries: are we preparing trainees appropriately to meet our needs?. Am J Surg.

[12] Nazem M, Beigi A-A, Mir-Mohammad Sadeghi A, Masoudpour H (2009). Non iatrogenic paediatric vascular trauma of the extremities and neck. Afr J Paediatr Surg.

[13] Allen CJ, Straker RJ, Tashiro J (2015). Pediatric vascular injury: experience of a level 1 trauma center. J Surg Res.

[14] (2019). Radial artery laceration. https://www.handsurgeryresource.com/radial-artery-laceration.

